# Anticancer Potential of Cucurbitaceae, Brassicaceae, Liliaceae and Chenopodiaceae: A Review of In Vitro Evidence

**DOI:** 10.3390/molecules31111902

**Published:** 2026-06-01

**Authors:** Edna C. Blanco-Torres, Gildardo Rivera, Timoteo Delgado-Maldonado, Eyra Ortiz-Pérez, Alma D. Paz-González, Ana Verónica Martínez-Vázquez, Erick de Jesús De Luna-Santillana, Jessica L. Ortega-Balleza, Lenci K. Vázquez-Jiménez

**Affiliations:** Laboratorio de Biotecnología Farmacéutica, Centro de Biotecnología Genómica, Instituto Politécnico Nacional, Reynosa 88710, Mexico; eblancot2400@alumno.ipn.mx (E.C.B.-T.); giriveras@ipn.mx (G.R.); titi_999@live.com (T.D.-M.); eortizp@ipn.mx (E.O.-P.); delpaz11@gmail.com (A.D.P.-G.); avmartinez@ipn.mx (A.V.M.-V.); edeluna@ipn.mx (E.d.J.D.L.-S.); jessica_ortega7@hotmail.com (J.L.O.-B.)

**Keywords:** cancer, natural products, anticancer activity, edible plants, nutraceuticals, cancer cell lines

## Abstract

Cancer remains a leading cause of death worldwide, and while various therapeutic strategies exist for its treatment, many have limited efficacy and significant adverse effects. Given this situation, there is a need to identify more effective and less aggressive therapeutic alternatives. In this context, natural products have garnered significant interest due to their potential as anticancer agents. This review aims to highlight the nutraceutical potential of edible plants belonging to the Cucurbitaceae, Brassicaceae, Liliaceae, and Chenopodiaceae families, evaluated in different tumor cell lines, as several studies have extensively demonstrated their antitumor activity and their potential application in cancer prevention and treatment.

## 1. Introduction

Cancer remains a leading cause of morbidity and mortality worldwide, representing a significant public health problem due to its high incidence, biological complexity, and therapeutic challenges [1]. Currently, traditional therapies such as chemotherapy, radiotherapy, and surgery continue to be the primary treatments. However, their limited efficacy and severe adverse effects have been demonstrated, especially in aggressive, resistant, or metastatic tumors. Chemotherapy, for example, often presents limitations in completely suppressing cancer cells, systemic toxicity, the development of drug resistance, and poor selectivity toward tumor cells [2]. Similarly, radiotherapy can induce damage to healthy tissues and, in some cases, contribute to the development of long-term adverse effects [3]. On the other hand, although surgery remains essential for eliminating the primary tumor, it is not always effective in the long term, as the loss of inhibitory factors from the tumor itself can stimulate the growth of previously inactive metastatic foci [4]. Taking these limitations highlights the need to identify new therapeutic and preventive alternatives that are more effective, safe, and accessible.

Natural products have garnered scientific interest due to their chemo preventive and therapeutic potential [5]. Edible plants represent an attractive source of bioactive compounds, as they are not only a nutritional value but also a common part of the human diet and could contribute to both cancer prevention and adjuvant treatment [6]. Within this group, various species belonging to the Cucurbitaceae, Brassicaceae, Liliaceae, and Chenopodiaceae families, which include widely consumed foods (Table 1), have attracted interest due to the growing experimental evidence that supports their biological activity. In this sense, the present review integrates the in vitro evidence on the anticancer potential of edible plants belonging to these four botanical families, simultaneously considering their biological activity, their mechanisms of action, and their possible nutraceutical value.

## 2. Cucurbitaceae Family

The Cucurbitaceae family includes several edible plants characterized by their rapid growth, with each plant producing numerous large fruits [7]. Furthermore, its species are distributed worldwide [8]. The Cucurbitaceae family represents the second largest family of fruits and vegetables [9,10], comprising approximately 130 genera and 800 species, including squash, cucumber, melon, and watermelon, among others, which are among the most important edible plants in the world [11,12].

### 2.1. Pumpkin

Pumpkin (*Cucurbita* spp.), and its different types of varieties (*C. moschata*, *C. pepo* L., *C. pepo* L. var, *C. maxima*, among others) commonly called pumpkin, squash, zucchini, etc. The global production of these plants was approximately 28 million metric tons, with China being the main producer with around 58% of world production [13,14]. In addition, their edible fruits, the seeds of these plants, have been traditionally used for nutrition and the relief of various diseases [15,16,17]. Several studies have demonstrated the anticancer activity of pumpkin extracts and their fractions against certain cancers, such as prostate, breast, leukemia, and melanoma [18,19,20,21]. Pumpkin seeds have also been investigated for their nutritional value and for producing high-quality oil, making them a source of protein [22,23]. Therefore, these compounds have garnered significant attention due to their nutritional and health-protective properties, as well as their pharmacological activity [24,25].

In this regard, Onuche and Abu [26] evaluated the preventive effect of including pumpkin leaves (*C. maxima*) on *N*-methyl-*N*-nitrosourea-induced colon carcinogenesis over 12 weeks. The authors observed a significantly lower level of carcinoembryonic antigen (CEA) in the serum of rats fed diets containing varying percentages of pumpkin leaves, compared to the control group (administered with *N*-methyl-*N*-nitrosourea without treatment), which exhibited high serum CEA levels (238.77 ng/mL). Furthermore, they observed no significant difference in malondialdehyde levels in the colon of the control group with a 10% dietary inclusion of *C. maxima* (52.08 nmol/mg/mL) compared to the control group. Histological analysis of colon tissue revealed severe damage to mucosal cells, with ulceration and sclerosis in the control group treated with *N*-methyl-*N*-nitrosourea. In contrast, the groups fed experimental diets and simultaneously administered *N*-methyl-*N*-nitrosourea showed only mild damage to normal cell architecture. These results demonstrated the ability of including pumpkin leaves in the diet to enhance the endogenous antioxidant system, reduce oxidative stress, and protect against organ damage induced by the carcinogen *N*-methyl-*N*-nitrosourea.

Biological studies of extracts and nanoparticles have also been conducted, highlighting pumpkins and their parts as potential antiproliferative agents (Table 2).

Table 2 shows that while several conventional extracts of leaves, seeds, or oil exhibited moderate or limited activity against different cell lines, some results were considerably more promising, such as the hydroalcoholic seed extract in papillary thyroid cancer. Additionally, nanoparticles biosynthesized from *Cucurbita* extracts significantly improved antitumor activity, showing effects on apoptosis, migration, cell adhesion, and the regulation of proteins associated with proliferation and the tumor stress response. Silver nanoparticles derived from the peel are particularly noteworthy, exhibiting high cytotoxicity and enhancing the effect of radiotherapy, suggesting a potential use as therapeutic adjuvants. However, these studies did not specify the cause of the increased activity of these nanoparticles, highlighting the need for further investigations into their mechanisms of action and the inclusion of more controls in biological assessments to determine the cause of the increased cytotoxic activity.

On the other hand, although most of these studies did not identify the metabolites present in the analyzed extracts, some authors identified unsaturated fatty acids, mainly oleic and linoleic acids, as well as palmitic acid in some oil extracts [29,30]. These metabolites have been previously associated with antioxidants, anti-inflammatory, and apoptosis-modulating activities, which could partially contribute to the observed effects. However, some studies suggest that anticancer activity does not depend solely on isolated compounds; for example, in the hydroethanolic seed extract, cucurbitin was not directly responsible for the antiproliferative effect, suggesting possible synergistic interactions among multiple metabolites present in complex extracts [28].

Another relevant aspect is the biological selectivity observed in several studies. Some extracts and nanoparticles showed lower toxicity on normal cell lines, such as fibroblasts, HUVEC, Vero, and primary liver cells, compared to cancer cells [27,28,30,31,34,39]. For example, silver nanoparticles obtained from the hydroethanolic fruit extract had a GI_50_ of 34.67 µg/mL in MCF-7 cells, while in HUVEC cells the GI_50_ was 70.46 µg/mL, indicating greater sensitivity in tumor cells [31]. Similarly, the hydroalcoholic leaf extract showed higher IC_50_ values in fibroblasts and CHO cells (239.2 and 241.4 µg/mL, respectively) than in HepG2 and CT26 cells (132.6 and 167.2 µg/mL, respectively) [27]. However, some oil extracts showed comparable toxicity between tumor and normal cells, which shows that selectivity still poses a challenge for certain formulations [30].

### 2.2. Cucumber

The cucumber (*Cucumis sativus* L.) is an important and economical crop with high water content and low-calorie count. It is consumed worldwide as a fruit in salads and as a vegetable [43,44,45]. China is the world’s leading producer, accounting for approximately three-quarters of total annual global production, with around 38 million tons produced in 2019 [46]. Furthermore, the anticancer potential of its seeds and isolates has been described [47,48]. Table 3 describes some of the most recent research demonstrating that cucumber has antiproliferative effects on cancer cells.

Table 3 shows that studies conducted with cucumber indicate that anticancer activity depends considerably on the type of formulation used and the chemical nature of the extracted metabolites. Silver nanoparticles derived from plant extracts exhibited significant cytotoxic activity in ovarian teratocarcinoma cells (IC_50_ of 49.71 µg/mL), associated with the induction of apoptosis as determined by flow cytometry analysis. In contrast, cucumber seed oils showed moderate activity against DU145 and PC3 cells, with inhibitions of approximately 70% at 100 µg/mL [12,50], accompanied by effects on apoptosis, migration, and cell invasion (determined by flow cytometry analysis) [12]. In addition to demonstrating a mechanism of action related to the reduction in proinflammatory cytokines such as IL-1β, IL-6, and TNF-α, as determined by enzyme immunoassays in both cancer cell lines [50].

The foamy cucumber extract showed an IC_50_ of 116.11 µg/mL against MDA-MB-231, indicating moderate activity associated with apoptosis (determined by flow cytometry analysis). In this case, cucurbitacins (A, B, C, and E) were identified along with glycosides such as gitoxigenin and digitoxigenin, metabolites previously associated with cytotoxicity and alteration of proliferative pathways [45].

On the other hand, the wild cucumber extract stood out for having one of the lowest IC_50_ values (7.5 µg/mL in A549), showing activity close to that of doxorubicin (6.0 µg/mL). However, the lack of phytochemical identification limits the ability to determine which compounds were responsible for the observed effect.

In terms of selectivity, most cucumber studies did not evaluate normal cell lines, making it difficult to establish a clear therapeutic index. This omission is a limitation, especially with nanoparticles, where high cytotoxicity could also be associated with damage to healthy cells. Therefore, although nanoparticles showed the greatest antiproliferative efficacy, further studies are still needed to determine their pharmacological applicability.

### 2.3. Watermelon

Watermelon (*C. lanatus*) is one of the best-known and most widely cultivated tropical fruits worldwide, representing 7% of global vegetable production [56]. Global watermelon production is approximately 79.2 billion tons, with China being the leading producer at 56.6 billion tons [57]. This fruit is a natural diuretic and is consumed fresh or as juice [58]. Although it is a widely consumed fruit worldwide, its anticancer potential, both in its rind and seeds, has not been fully explored. Potential free radical scavenging, antioxidant, and antimicrobial activity have been found in the seeds, along with a potential reduction in the risk of cardiovascular disease and cancer [53,58,59].

Among these, the study by Elhassaneen et al. [60] stands out. They obtained an ethanolic extract of watermelon rind and evaluated its protective effect on rat liver cell cultures with molecular damage induced by benzo[*a*]pyrene. The results showed that the watermelon rind extract reduces benzo[*a*]pyrene-induced cytotoxicity. Exposure to 10 µM of benzo[*a*]pyrene caused adverse effects in liver cells, with 84.5% cell death. However, the administration of watermelon rind extracts reduced cytotoxicity to liver cells; at a concentration of 75 µg/mL, the percentage of dead cells was reduced to 40%. These findings demonstrate the potential of watermelon rind extract to prevent molecular damage and improve cell viability.

Table 3 shows other studies on watermelon rinds and seeds that demonstrate that both extracts and nanoparticles possess anticancer activity, although with important differences in potency, mechanisms of action, and selectivity. In general, nanoparticles obtained from the seed extract showed greater cytotoxic than crude extracts, particularly against HCT-116 and HepG2 cells. For example, the nanoparticles achieved IC_50_ values of 33.7 and 44.0 µL, respectively, while the seed extract without nanoparticles showed less potent IC_50_ values in the same cell lines (40.1 and 79.2 µL, respectively) [52]. In contrast, the pee extracts exhibited greater activity, with IC_50_ values of 20–24 µg/mL against HCT-116 and HepG2, respectively. This activity was associated with the induction of apoptosis, cell cycle arrest in S phase, and the regulation of markers such as caspase-3 and the BAX/BCL-2 ratio (determined by cell cycle analysis, flow cytometry, and gene expression levels). This activity could be related to the abundance of phenolic compounds, flavonoids, coumarins, and stilbenes identified in the peel, metabolites widely associated with antioxidant properties and cell signal modulation [53].

The peel extract also showed interesting effects on HRAC-769-P adenocarcinoma, although much higher concentrations (156.8 mg/mL) were required to achieve inhibitions close to 66%. Despite this lower potency, early polycaspase responses and upregulation of genes associated with intrinsic and extrinsic apoptosis were observed, as determined by transcriptomic analysis [54].

Regarding the solvents, the aqueous and ethanolic extracts showed distinct profiles. The ethanolic seed extract had an IC_50_ of 51.73 µg/mL against A549 and induced cell cycle arrest in the G2 phase and apoptosis (determined by cell cycle analysis) [55]. Ethanolic extraction likely favored the recovery of phenolic compounds, tannins, flavonoids, and saponins, all identified in the study. Furthermore, this extract showed high biological selectivity, producing only 0.62% inhibition in Vero cells at 125 µg/mL, indicating a marked preference for tumor cells [55].

### 2.4. Melon

*Cucumis melo* (L.) is the scientific name for the melon, which is cultivated in temperate, subtropical, and tropical regions worldwide, with a global production of 27.5 million metric tons in 2019 [61,62,63]. China is the leading melon producer, with a production of 17.1 million metric tons [64]. Traditionally, it has been reported to possess antioxidant, anti-inflammatory, immunomodulatory, hepatoprotective, and anticancer activity [65,66]. However, there are still a few scientific studies on the anticancer effects of melon. Among these few studies, Vidya et al. [63] explored the anticancer efficacy of silver nanoparticles derived from aqueous melon extract on Wistar rats in which liver cancer was induced with 0.01% diethylnitrosamine via their drinking water intake for 16 weeks. The anticancer activity of the silver nanoparticles from aqueous melon extract was similar to that in rats treated with cyclophosphamide. They also found that the silver nanoparticles from aqueous melon extract exhibited the chemotherapeutic effect only in tumor cells, but not in normal cells, as revealed by toxicity studies (markers of liver function and histological study of liver and kidney tissue). Confirming the anticancer potential of silver nanoparticles from melon could be due to the additive and synergistic effects of melon secondary metabolites.

## 3. Brassicaceae Family

The Brassicaceae family is one of the best known because it contains some of the most consumed cruciferous vegetables worldwide, with a total production of almost 73 kilotons in 2022 [67]. Furthermore, this family has been described as providing significant health benefits, such as anticancer properties [68,69,70]. Consumption of Brassicaceae has been associated with a reduced risk of common cancers such as breast cancer [71], prostate cancer [72], bladder cancer, colon cancer, pancreatic cancer [73], and colorectal cancer [74].

### 3.1. Radish

The radish (*Raphanus sativus* L.) is a root vegetable that occupies a prominent place in the global diet, with an annual production of approximately 7 million tons, representing 2% of global vegetable production [75,76]. In traditional medicine, extracts from the green and underground parts of the radish have been used to treat various liver, cardiovascular, and gastric disorders, as well as urinary tract infections [77]. Some studies have been conducted on its use in cancer, as described in Table 4.

In Table 4, shows that radish studies exhibit wide variability in cytotoxic activity depending on the type of extract, the solvent used, and the nanoparticles employed. The hexane root extract stands out for its high cytotoxic potency, with IC_50_ values between 7 and 10 μg/mL against various cell lines (HeLa, A549, and MCF-7), while it showed lower sensitivity against the PC-3 cell line (IC_50_ between 12 and 21 μg/mL). These values were comparable to etoposide, which presented IC_50_ values between 10 and 22 μg/mL.This activity was also associated with the induction of apoptosis and differential regulation of genes in the Bcl-2 family, favoring the overexpression of pro-apoptotic genes and decreasing anti-apoptotic genes. The high activity observed in the hexane extract is likely associated with the presence of isothiocyanates identified by GC-MS, including 4-(methylthio)-3-butenyl isothiocyanate, erucine, sulforaphene, and other bioactive sulfur compounds. Glucosinolate-derived isothiocyanates are known to modulate oxidative stress, apoptosis, and cell cycle arrest, which partially explains the high cytotoxicity observed [78].

In contrast, other conventional root and leaf extracts showed more moderate or limited activity (IC_50_ values greater than 200–400 μg/mL). These extracts primarily contained phenolic compounds and flavonoids, such as gallic acid, quercetin, and rutin, metabolites frequently associated with antioxidant and antiproliferative activities [80]. Some studies have also demonstrated specific molecular mechanisms. The ethanolic leaf extract inhibited MDA-MB-231 cell proliferation by regulating the ErbB-Akt pathway, decreasing the expression of ErbB2, ErbB3, and Akt, and increasing Bax (determined by Western blot analysis, protein expression analysis, and mRNA analysis). This indicates that the extracts can act on both cell survival and apoptosis [79].

Importantly, radish-derived nanoparticles, especially zinc oxide nanoparticles and reduced graphene oxide, significantly improved biological activity in certain cell lines [81,82]. Zinc oxide nanoparticles obtained from leaves showed an IC_50_ of 8.05 μg/mL compared to MCF-7, much higher than the plant extract alone (IC_50_ = 95.43 μg/mL) and doxorubicin (IC_50_ = 3.33 μg/mL). While its phytochemical analysis showed the presence of phenols, flavonoids, alkaloids, and tannins [82], similarly, root-derived zinc oxide nanoparticles showed selective cytotoxicity against A549 (IC_50_ = 22.79 μg/mL), whereas in normal MCF-10 cells the IC_50_ was 272.24 μg/mL, demonstrating marked therapeutic selectivity [84]. This provides evidence of mechanisms such as apoptosis (determined by programmed cell death assay and cell cycle analysis) [82], the induction of autophagy and the modulation of inflammatory processes (determined by apoptosis assay, immunofluorescence, enzymatic assay, and flow cytometry for the detection of autophagy and phagocytosis) [84].

### 3.2. Kale

Kale, also known as curly kale (*Brassica oleracea* var.), is an important crop in agriculture due to its ease of cultivation, low cost, and resistance to harsh environmental conditions [91]. Although global kale production is difficult to estimate since it is often grouped with other brassicas, approximately 124,000,000 pounds of kale were sold in the United States in 2017 [92]. Some cancer research has also been conducted on kale, as described in Table 4. For example, crude kale extracts exhibited moderate cytotoxic effects in the A549 and HeLa cell lines [85,87], while in other models, such as U937 and HepG2 [88,89], higher inhibition percentages were observed, suggesting a cellular response dependent on the cancer type. Ferulic acid, sinapic acid, salicylic acid, kaempferol, and quercetin were identified in the leaf extract by LC-MS/MS [87]. While studies of kale using natural juice and steamed kale determined that the cooking method does not alter the levels of total glucosinolates and isothiocyanates [88], nor does the variety interfere with the content of the latter, observing apoptosis mediated by caspase-3 activation as the mechanism of action, although without significant alterations in Bax and Bcl-2 (determined by flow cytometry and Apoptotic Protein Expression) [89].

Studies conducted with kale showed moderate to high cytotoxic activities depending on the variety, the preparation method, and the type of extract used. The ethanolic extract against PC3 cells presented an IC_50_ of 351.7 μg/mL, while in normal RWPE-1 cells the IC_50_ was 702.3 μg/mL, demonstrating a certain degree of selectivity towards prostate tumor cells. The mechanism of action involved the induction of apoptosis through the upregulation of pro-apoptotic genes such as BAX and activation of the NRF2 pathway, along with the downregulation of inflammatory and cell survival genes such as NF-κB, BCL-2 (determined by qRT-PCR, DNA fragmentation assay, and Western blot). This supports the idea that kale not only exerts cytotoxicity but also has a possible effect on mechanisms involved in tumor survival, inflammation, and progression. LC-MS/MS analyses identified a wide variety of bioactive metabolites, including isothiocyanates (sulforaphane, erucine, and isobutyl isothiocyanate), glucosinolates (sinigrin and glucoiberverine), phenolic compounds (sinapic, caffeic, ferulic, and chlorogenic acids), and flavonoids such as quercetin, kaempferol, and rutin. Many of these compounds have recognized chemopreventive effects, particularly sulforaphane and indole-3-carbinol, which regulate cellular detoxification, apoptosis, and cell cycle arrest [86].

Another interesting study involved shoots treated with γ-polyglutamic acid, whose methanolic extracts showed IC_50_ values between 130 and 153 μg/mL against HCT116. These extracts did not exhibit an inhibitory effect on normal cells (CCD 841 CoN), with approximately 100% survival, suggesting high selectivity [90].

Overall, the results demonstrate that kale possesses multifactorial anticancer activity primarily associated with glucosinolates, isothiocyanates, and phenolic compounds. Although some extracts show moderate potency, several studies demonstrate relevant molecular mechanisms related to apoptosis, redox regulation, and suppression of inflammatory pathways, as well as some selectivity toward tumor cells.

### 3.3. Arugula

Arugula (*Eruca sativa* Mill.) is commonly used in salads [93]. Arugula leaves are considered an important source of nutrition due to the presence of various nutrients [94].

Furthermore, some studies have been conducted on the anticancer properties of extracts from parts of arugula in cell lines (Table 5). The results show an anticancer potential associated with their isothiocyanate and phenolic compound content. Cytotoxic activity varied depending on the type of extract and the nanoparticle used. The seed oil exhibited moderate cytotoxicity against murine melanoma cells (B16F10) and human melanoma cells (MDA-MB-435), with IC_50_ values of 24.78 and 34.45 μg/mL, respectively. Phytochemical analysis by U-HPLC-ESI-MS3 identified several bioactive isothiocyanates, including allyl isothiocyanate, 3-butenyl isothiocyanate, 2-phenylethyl isothiocyanate, sulforaphane, and erucine. These metabolites are widely recognized for inducing apoptosis, modulating detoxification enzymes, and altering pathways related to cell proliferation and oxidative stress, which likely explains the observed activity [95].

The ethanolic leaf extract showed relevant activity against the MCF-7 cell line (IC_50_ = 41.13 μg/mL), associated with apoptosis and cell cycle arrest in G2/M phases, as determined by flow cytometry with Annexin V-FITC. These results suggest that the extract may interfere with cell progression and activate programmed cell death mechanisms [96]. In HCT-116 and Caco-2 cells, the ethanolic extracts showed IC_50_ values of 64.91 and 83.98 μg/mL, respectively. HR-LC/MS analysis revealed a highly complex chemical composition, including flavonoids, terpenoids, alkaloids, fatty acids, phenols, and indole derivatives. The simultaneous presence of multiple bioactive metabolites suggests possible synergistic effects responsible for the observed antiproliferative activity [97].

Silver nanoparticles synthesized from leaf extract showed a significant improvement in cytotoxic activity compared to A549 (IC_50_ = 25.15 μg/mL). Furthermore, inhibition of cell migration was observed in invasion assays, indicating a possible antimetastatic effect. GC-MS analysis identified compounds such as sulforaphane nitrile, phytol, and octadecatrienoic acid, metabolites associated with antioxidant, apoptotic, and anti-inflammatory activities [98].

Studies have also been carried out in in vivo models, such as the study carried out by Khoobchandani et al. in 2011 [95], where seed oil significantly inhibited melanoma tumor growth in C57BL/6 mice inoculated with B16F10 cells, by 19.79% and 29.48% at doses of 1 and 2 mg/kg of body weight, respectively.

**Table 5:** In vitro anticancer activity of extracts, oil, and nanoparticles derived from cruciferous plants (arugula, watercress, and broccoli).

Product Used	Cell Line	In Vitro Activity	Mechanism of Action	Reference
**Arugula (*Eruca sativa* Mill.)**				
Seed oil	B16F10 (Murine melanoma)	IC_50_ = 24.78 μg/mL	ND	[95]
	MDA-MB-435 (human melanoma)	IC_50_ = 34.45 μg/mL		
Methanolic extract biofertilized with 10% seaweed extract	HepG2	IC_50_ = 85.7 μg mL^−1^	ND	[99]
Ethanolic leaf extract	MCF-7	IC_50_ = 41.13 µg/mL at 48 h	Apoptosis and inhibition produced in the G2 and M phases of the cell cycle	[96]
Ethanolic leaf extract	HCT-116	IC_50_ = 64.91 µg/mL	ND	[97]
	Caco-2	IC_50_ = 83.98 µg/mL		
Silver nanoparticles from leaf extract	A549	IC_50_ = 25.15 µg/mL at 24 h	Inhibition of cancer cell migration	[100]
**Watercress (*Nasturtium officinale* L.)**				
Polyethylene glycol and poly(lactic-co-glycolic acid) (PEG-PLGA) nanoparticles encapsulated with watercress extract	A549	IC_50_ = 58.2 µg/mL at 24 h	Apoptosis and significantly increased expression of p53, Bax and Caspase 3	[98]
		IC_50_ = 40.4 µg/mL at 48 h		
		IC_50_ = 35.0 µg/mL at 72 h		
Freeze-dried powder from watercress	A375 (Malignant melanoma)	EC_50_ = 9.99 μM at 24 h	Induction of caspase-9 and -3 levels, compared to caspase-8, indicates apoptosis via the intrinsic mitochondria-dependent pathway	[101]
		EC_50_ = 2.48 μM at 48 h		
		EC_50_ = 1.71 μM at 72 h		
	A431 (Non-melanoma epidermoid carcinoma)	EC_50_ = 37.29 μM at 24 h		
		EC_50_ = 15.28 μM at 48 h		
		EC_50_ = 17.42 μM at 72 h		
Aqueous leaf extract	OCC-24 (Oral cancer)	IC_50_ = 2.12 µg/mL at 24 h	ND	[102]
		IC_50_ = 3.83 µg/mL at 48 h		
Gold nanoparticles from the extract	A549	IC_50_ = 39.84 µg/mL at 24 h	Apoptosis	[103]
		IC_50_ = 25.05 µg/mL at 48 h		
**Broccoli (*Brassica oleracea* L. *italica*)**				
Aqueous extract	A549	Inhibition = 39% at 500 μg mL^−1^ at 72 h	ND	[85]
80% alcoholic extract	HCT116	IC_50_ = 3.88 µg/mL	ND	[104]
70% ethanolic extract	HepG2	IC_50_ = 0.11 µg/mL at 48 h	Apoptosis through an increase in the percentage of cells in the subG1 phase and loss of mitochondrial membrane potential	[105]
	Caco-2	IC_50_ = 0.16 µg/mL at 48 h		
	A549	IC_50_ = 0.18 µg/mL at 48 h		
Chloroform fraction of the methanolic extract of floret	MCF7/SC (Breast cancer)	IC_50_ = 69.47 µg/mL	Suppression of pluripotency characteristics and induction of apoptotic cell death	[106]
Hexane fraction of the methanolic extract of floret		IC_50_ = 81.53 µg/mL	ND	

ND: Not determined.

### 3.4. Watercress

Watercress (*Nasturtium officinale* L.) is a fast-growing, annual aquatic or semi-aquatic plant cultivated in temperate climates worldwide, with an annual global production of approximately 15,000 tons [107,108]. The most important producers of watercress are India with 60% of production, Ethiopia with 20%, and Pakistan with 15% [109]. Its anticancer pharmacological activity has been investigated (Table 5) [107,108].

Table 5 shows that watercress exhibits more consistent and potent anticancer activity, especially in models of melanoma, lung, and oral cancer. A clear time-dependent relationship is observed, with IC_50_/EC_50_ values decreasing with longer exposure times. Furthermore, several studies report mechanisms of action, primarily the induction of apoptosis via the intrinsic mitochondrial pathway (caspase activation). Its use as nanoparticles also enhances its efficacy, reinforcing its therapeutic potential.

PEG-PLGA nanoparticles encapsulated with watercress extract exhibited time-dependent activity against A549, reaching an IC_50_ of 35.0 μg/mL at 72 h. The mechanism of action involved apoptosis mediated by a significant increase in p53, Bax, and caspase-3, indicating activation of mitochondrial apoptotic pathways. Furthermore, the nanoparticles showed greater cytotoxicity than the free extract [98]. Gold nanoparticles synthesized from the extract also showed activity against A549, associated with apoptosis and cell cycle inhibition as determined by flow cytometry. Moreover, the nanoparticles showed greater inhibition than the plant extract alone (24% inhibition at 48 h) and then the nanoparticles without the extract (0% inhibition at 48 h) [103].

Furthermore, freeze-dried cress powder exhibited particularly high activity against malignant melanoma (A375), with an EC_50_ of 1.71 μM at 72 h. It also showed significant selectivity against non-tumor HaCaT cells, whose EC_50_ values were considerably higher (38.92 μM at 72 h). The mechanism involved activation of caspases-9 and -3, suggesting apoptosis mediated by the intrinsic mitochondrial pathway [101]. Phytochemical analysis by UPLC-MS/MS indicated that the sample was rich in isothiocyanates, polyphenols, phenolic compounds, β-carotene, lycopene, and ascorbic acid. Among these metabolites, phenethyl isothiocyanate stands out for its recognized anticancer activity related to apoptosis, proliferative inhibition, and redox modulation [101].

The aqueous leaf extract also showed high activity against OCC-24 oral cancer cells, with an IC_50_ of 2.12 μg/mL at 24 h, demonstrating a potent antiproliferative capacity even without the use of nanoparticles [102].

Furthermore, watercress leaf extract has also been used to evaluate its hepatoprotective effect on *N*-diethylnitrosamine-induced hepatocellular carcinoma in male Wistar rats. An LD_50_ of 707.2 mg/kg was determined for the cress leaf extract, suggesting that lower doses would be safe for further studies. Furthermore, they observed that administering *N*-diethylnitrosamine reduced body weight and increased liver weight; however, the cress leaf extract at 200 mg/kg significantly increased the rats body weight and decreased liver weight. They also demonstrated that administering *N*-diethylnitrosamine significantly increased levels of pro-inflammatory cytokines such as TNF-α, IL-1β, and IL-6 and decreased levels of the cytokine IL-10, while the cress leaf extract at 200 mg/kg increased serum IL-10 levels and decreased levels of pro-inflammatory cytokines. Additionally, watercress extract restored liver lobes to a normal state, reduced lymphocytic infiltration, and eliminated liver nodules, thus highlighting the potential of watercress leaf extract to exert anticancer effects [110].

### 3.5. Broccoli

Broccoli (*Brassica oleracea* L. *italica*) is a green plant with large flowers, used as a vegetable [111]. The florets are the most consumed part and constitute 30% of the whole broccoli [112]. Global annual broccoli production was 27.46 million tons in 2020. China is the world’s largest producer and consumer of broccoli, producing more than 1 million tons [113]. This vegetable has been marketed as a health food and has been described as having the ability to act as a preventative agent against various types of cancer and other disorders [114,115].

For several decades, the use of broccoli by products, such as leaves and stems, has been limited to flour and fiber [112], but the potential use of these byproducts as important sources of health-promoting phytochemicals has begun to be investigated [116]. In this regard, Hwang et al. [117] evaluated the anticancer activities of 80% methanol extracts of different byproducts from various cultivars (Kyoyoshi, Myeongil 96, and SK3-085) and harvest dates against NCI-H1299 cell lines (human non-small cell lung carcinoma) using the MTT (3-(4,5-dimethylthiazol-2-yl)-2,5-diphenyltetrazolium bromide) assay. Extracts of florets, leaf stalks, and stems from the Kyoyoshi culture showed greater inhibitory activity than those from Myeongil 96 and SK3-085 against NCI-H1299 cell lines. The leaf extract from Myeongil 96 (32.5%) exhibited the highest cell growth inhibition activity against the NCI-H1299 cell lines compared to all other byproducts. Specifically, the leaves from all cultivars showed the highest cell growth inhibition activities, in decreasing order of florets, leaf stalks, and stems. The results of this study demonstrate that broccoli leaves and stems offer potential as functional fresh raw vegetables.

Furthermore, edible florets are believed to be high in phytochemicals and promote health; they are also known to prevent some chronic degenerative diseases [118]. Among these, broccoli florets (*Brassica oleracea* L. var. *italica*) have gained recognition as nutraceutical foods due to their contribution to the prevention of chronic, cardiovascular, and cancerous diseases [119,120]. Table 5 shows some examples.

Among the three plants presented in Table 5, broccoli stands out for its high cytotoxic potency, with lower IC_50_ values in some cell lines (particularly liver, colon, and lung). Its ethanolic extracts show a strong capacity to induce apoptosis, associated with cell cycle alterations and mitochondrial dysfunction. While certain less polar extracts may exhibit reduced activity, broccoli consistently demonstrates high anticancer potential.

The aqueous extract achieved 39% inhibition at 500 μg/mL. Standard sulforaphane showed greater inhibition (69%), while an evaluation of the sulforaphane-enriched aqueous extract showed a positive correlation between sulforaphane content and cytotoxicity against A549, as described by the authors [85]. The 80% alcoholic extract showed potent activity against HCT116, with an IC_50_ of 3.88 μg/mL, while the reference drug cisplatin showed no effect. Glucosinolates such as glucoiberin and bioactive sulfur derivatives were identified by LC-ESI [104].

One of the most notable results was obtained with the 70% ethanolic extract, which showed low IC_50_ values against HepG2, Caco-2, and A549 (0.11–0.18 μg/mL). Furthermore, it exhibited selectivity against normal liver cells (FL83B) with an IC_50_ > 0.500 μg/mL. The mechanism of action involved apoptosis associated with an increase in subG1 cells and loss of mitochondrial membrane potential, indicating activation of the intrinsic apoptotic pathway as determined by flow cytometry. HPLC analysis identified high concentrations of gallic acid, caffeic acid, ferulic acid, quercetin, and myricetin [105].

The methanolic extract fractions of florets also showed activity against MCF7/SC breast cancer stem cells. The chloroform fraction (IC_50_ = 69.47 µg/mL) showed greater activity than the hexane fraction (IC_50_ = 81.53 µg/mL) and was able to suppress pluripotency and induce apoptosis (determined by flow cytometry and a Western blot assay), which is particularly relevant due to the role of tumor stem cells in recurrence and therapeutic resistance. Furthermore, when they analyzed the crude methanol extract, they observed that the most abundant phenolic compounds were rutin, quercetin, chlorogenic acid, catechin, and *p*-coumaric acid [106].

### 3.6. Brussels Sprouts

Brucella sprouts (*Brassica oleracea* var. *gemmifera*), also known as Brussels cabbage, are among the oldest cultivated crops in the world and have become an important crop in global agricultural production. They are intensively cultivated in Belgium, occupying 380 hectares of land in Flanders in 2021, with 8740 tons of Brussels sprouts available for sale as fresh produce [121]. Although few studies have described its anticancer properties [122,123], some research has been published on the effect of Brussels sprout extracts on lung and breast cancer (Table 6).

The data available in Table 6 for Brussels sprouts indicates moderate antiproliferative activity; however, the evidence reported to date remains limited. Against the A549 cell line, the aqueous extract showed relatively low inhibition (28% against A549 cells at 500 μg/mL after 72 h) compared to the sulforaphane standard, which achieved 69% inhibition under these experimental conditions. GC-MS analysis revealed a high sulforaphane content, suggesting that this isothiocyanate contributes significantly to the observed activity. However, the lower cytotoxicity of the whole extract could be due to lower bioavailability or effective concentration of the active compound within the plant matrix [85]. In contrast, the ethanolic extract evaluated against MDA-MB-231 cells showed an IC_50_ value of 210.41 µg/mL, demonstrating more defined activity and suggesting that the nature of the extraction solvent influences the recovery of bioactive compounds. Phytochemical analysis indicated the presence of saponins, phenols, alkaloids, flavonoids, glycosides, and amides [123].

Taken together, these findings suggest that Brussels sprouts possess in vitro anticancer potential, although apparently less than that observed in other Brassicaceae included in Table 6. Furthermore, the lack of mechanistic information limits the biological interpretation of its effects, so further studies are needed to clarify the cellular mechanisms involved, particularly those related to apoptosis, oxidative stress, and cell cycle regulation.

### 3.7. Cabbage

Red cabbage (*Brassica oleracea* var. *capitata*) is a cruciferous vegetable popular worldwide and attractive to consumers due to its nutritional value [125,128]. According to the FAO, cabbage represents an annual global production of 71.8 million tons, with China being the leading producer at 33.9 million tons [68]. Recently, cabbage has attracted attention due to its potential anticancer effects [129,130].

In this regard, Table 6 presents the antiproliferative effects of different cabbage extracts against various cancer cell lines, highlighting their anticancer potential. The results show that different types of extracts exert cytotoxic effects on various tumor cell lines, suggesting a broad spectrum of activity in vitro. Specifically, cabbage extracts and their phenolic fractions showed the lowest IC_50_ values. The variability observed among aqueous, alcoholic, methanolic, and phenolic extracts also confirms that the extraction method influences biological potency. Taken together, these findings position cabbage as one of the Brassicaceae with the greatest anticancer potential within the studies compared in Table 6.

For example, the aqueous extract showed 63% inhibition against A549 and an IC_50_ of 38 μg/mL, demonstrating superior activity compared to other Brassicaceae evaluated. Furthermore, the authors describe a positive correlation they observed between sulforaphane content and the cytotoxicity of organic extracts enriched with this compound, confirming sulforaphane’s central role as an anticancer agent [85]. One of the most relevant results was observed with the cabbage extract against HeLa and HepG2, with IC_50_ values of 23.38 and 28.66 μg/mL, respectively. This extract also showed significant biological selectivity, as the CC_50_ in normal PBMC cells was 251.28 μg/mL, yielding selectivity indices of 10.88 for HeLa and 8.93 for HepG2. The mechanism of action involved apoptosis, increased TNFα, and cell cycle arrest in the G0/G1 phase, determined by flow cytometry, ELISA, and real-time RT-PCR. These findings indicate that the extract can modulate both cell proliferation and cancer-associated inflammatory signals [124].

Furthermore, juices from young shoots exhibited antiproliferative activity against DU145 and LNCaP compared to mature plants. This was linked to the higher concentrations of vitamin C, flavonoids, anthocyanins, and phenolic compounds found in young shoots, suggesting that the plant’s developmental stage significantly influences its phytochemical composition and biological activity [125]. Alcoholic and phenolic leaf extracts also showed significant activity against HeLa, MCF-7, and HepG2, with the phenolic extract exhibiting a notable IC_50_ of 17.71 μg/mL against HeLa. UPLC-PDA-ESI-qTOF-MS analysis identified multiple cyanidin-derived anthocyanins and several kaempferol derivatives [126].

### 3.8. Cauliflower

Cauliflower (*Brassica oleracea* var. *botrytis*) is a vegetable with a global production of 25.50 million metric tons in 2020. Among global producers, China is the leading producer with 10.3 million tons [67,131]. It has also been described in studies with anticancer effects [132]. Table 6 shows the activity of aqueous extracts with antiproliferative potential. The aqueous extract of purple cauliflower showed inhibition against HT29 (75% at 24 h), while the white cauliflower extract showed only 25% inhibition under the same conditions. Phytochemical analysis by HPLC-DAD-MS/MS identified various bioactive glucosinolates and isothiocyanates, including glucoiberin, glucobrassicin, glucoraphanin, and sinigrin, as well as isothiocyanates such as 3-methylsulfinylpropyl isothiocyanate, sulforaphane, and allyl isothiocyanate. Indolic compounds such as indole-3-carbinol, indole-3-acetic acid, indole-3-acetonitrile, and diindolylmethane were also identified. Among the phenolic acids identified, sinapic, *p*-coumaric, and vanillic acids were prominent in both varieties. However, the authors noted that no direct correlation was found between biological activity and the content of anthocyanins or glucosinolate derivatives, indicating that the anticancer activity likely depends on complex interactions among multiple metabolites [127].

Research has also been conducted to evaluate its potential as a chemopreventive agent using juice obtained from cauliflower leaves on breast cancer cell lines (MCF-7, BT474, MDA-MB-231, and BT20), demonstrating that cauliflower juice suppressed cell proliferation in a dose-dependent manner and reduced DNA synthesis. Inhibition of cell growth was accompanied by significant cell death at the highest juice concentrations, although no evidence of apoptosis was found. Furthermore, the inhibition of proliferation was associated with significantly reduced CDK6 expression and increased p27 levels in MCF-7 and BT474 cells, but not in MDA-MB-231 and BT20 cells, while significantly decreased phosphorylation of the retinoblastoma protein was observed in all cell lines [133].

### 3.9. Summary and General Perspectives

The studies analyzed in the Brassicaceae family clearly demonstrate that solvent polarity can influence the recovery of bioactive compounds and, consequently, the observed anticancer activity. In general, polar solvents such as water, methanol, and ethanol favored the extraction of phenolic acids, flavonoids, anthocyanins, and glucosinolates, while less polar solvents such as hexane and chloroform preferentially recovered lipophilic sulfur-containing compounds and isothiocyanates. These differences directly impacted cytotoxic potency and selectivity. For example, the hexane extract of radish roots exhibited one of the highest cytotoxic activities reported, with IC_50_ values between 7 and 10 μg/mL in several cancer cell lines. This high activity was associated with sulfur-containing isothiocyanates, such as sulforaphene, erucine, and 4-(methylthio)-3-butenyl isothiocyanate, compounds with relatively hydrophobic characteristics that are more efficiently extracted using nonpolar solvents. In contrast, aqueous and ethanolic extracts of other Brassicaceae species frequently showed moderate activity but contained phenolic compounds and flavonoids with recognized antioxidant, anti-inflammatory, and chemopreventive properties. Furthermore, the greater activity of the chloroform fraction of broccoli florets compared to the hexane fraction further supports the importance of intermediate-polarity solvents for concentrating bioactive metabolites with antiproliferative effects.

The comparison between species suggests that broccoli, watercress, radish, and cabbage exhibited the most consistent and potent anticancer activities. Broccoli demonstrated low IC_50_ values in liver, colon, and lung cancer models, along with clear apoptotic mechanisms and selectivity toward tumor cells. Watercress also stood out for its potent activity against melanoma, lung, and oral cancer cells, in addition to significant evidence of apoptosis mediated through intrinsic mitochondrial pathways and favorable selectivity toward normal cells. Radish showed remarkable activity, especially in nanoparticles and nonpolar extracts enriched with isothiocyanates. Cabbage, for its part, exhibited broad-spectrum activity with high selectivity indices and modulation of inflammatory and apoptotic pathways. In contrast, Brussels sprouts and some kale extracts generally showed more moderate activity, although they demonstrated chemopreventive potential, possibly due to their glucosinolate and phenolic compound composition.

Another important aspect that emerges from these studies is the growing relevance of nanoparticles. Zinc oxide, silver, gold, graphene oxide, and PEG-PLGA nanoparticles frequently enhanced cytotoxic activity, improved selectivity, and enabled additional biological effects. However, these findings only suggest that these nanoparticles may improve the bioavailability, stability, and cellular uptake of Brassicaceae-derived phytochemicals, thereby increasing their therapeutic potential. However, since most of the studies presented in this review are preliminary, further biological analyses are needed to confirm these inferences.

Despite these results, many studies still lack detailed mechanisms of action analyses. Therefore, future research should incorporate omics approaches, including transcriptomics, proteomics, metabolomics, and epigenomics, to identify the molecular pathways involved in the anticancer effects of Brassicaceae extracts and nanoparticles. Integrative omics analyses would allow the identification of key signaling networks related to apoptosis, oxidative stress, inflammation, autophagy, immune modulation, and cell cycle regulation. Furthermore, metabolomic profiling combined with bioinformatics and systems biology could help determine which specific metabolites or synergistic combinations of metabolites are responsible for the observed biological activities.

In addition, future research could expand the evaluation of synergistic interactions between Brassicaceae-derived extracts and conventional or targeted anticancer therapies. Several phytochemicals identified in these vegetables, particularly sulforaphane, phenethyl isothiocyanate, quercetin, kaempferol, and indole-3-carbinol, have been associated with chemosensitization, reduction in oxidative damage, inhibition of multidrug resistance pathways, and enhancement of apoptosis. Therefore, combination therapies could reduce the effective dose of chemotherapeutic agents, minimize toxicity and overcome resistance mechanisms.

Furthermore, from a nutraceutical perspective, vegetables of the Brassicaceae family represent important dietary sources of bioactive phytochemicals with potential chemopreventive properties. Vegetables rich in sulforaphane, such as broccoli, watercress, radish, and cabbage, could be promoted as functional foods due to their ability to regulate detoxification enzymes, induce apoptosis, and reduce inflammation. Furthermore, red cabbage and purple cauliflower, rich in anthocyanins, may provide additional antioxidant and anti-inflammatory benefits. These findings support dietary recommendations that encourage the regular consumption of cruciferous vegetables as part of cancer prevention strategies. Future nutraceutical research could focus on standardizing the content of bioactive phytochemicals, evaluating bioavailability after cooking and digestion, and developing functional foods or supplements enriched with specific glucosinolates, isothiocyanates, and phenolic compounds.

Overall, the available evidence indicates that Brassicaceae species are promising sources of anticancer bioactive phytochemicals with potential applications in the development of nutraceuticals, adjuvant therapies, or nanomedicine. However, further standardized studies integrating mechanism of action analysis, omics technologies, in vivo validation, and clinical evaluation are needed to fully establish their therapeutic relevance.

## 4. Liliaceae Family

In this family, the genus *Allium* comprises more than 500 species, including *A. cepa* L. (onion), *A. sativum* L. (garlic), *A. schoenoprasum* L. (chives), and *A. ampeloprasum* (leek) [134,135,136]. These vegetables are widely consumed as spices for their distinctive flavors and health benefits [137]. It has been described that those consuming vegetables from the lily family inhibit some types of cancer, such as stomach, colorectal, and prostate cancer [138].

### 4.1. Onion

The onion (*Allium cepa* L.) is a staple food consumed worldwide, with China being the largest producer at nearly 24 million tons [139]. Onions have been described as having positive health effects associated with their consumption due to their high levels of antioxidant compounds, which offer protection against various chronic degenerative diseases such as cancer [140,141]. Table 7 describes some studies that have evaluated onions and parts of the plant against various cancer cell lines, including lungs, breast, and pancreatic cancer, among others, providing new perspectives on the potential use of onion derivatives for cancer treatment.

The results shown in Table 7 demonstrate that conventional extracts exhibit moderate activity, with relatively high inhibition percentages and IC_50_ values in some cell lines. The peel extract showed activity against HT-29 cells, with 62.93% inhibition at 100 µg/mL. However, the study also reported high inhibition in normal HUVECs (69.97%), suggesting low selectivity and possible nonspecific toxicity. The mechanism of action included apoptosis mediated by downregulation of L1CAM-associated pathways, as well as inhibition of cell migration and invasion (determined by Transwell wound healing and invasion assays, and indirect immunofluorescence) [144].

The onion extract evaluated in Caco-2 cells showed approximately 55% inhibition at high concentrations (1000 µg/mL). The mechanism involved S-phase cell cycle arrest, a decrease in G1 cells, and activation of p53 and caspase-3 (determined by flow cytometry), further confirming the involvement of apoptosis and cell cycle regulation. Additionally, several phenolic compounds present in the extracts were identified by HPLC-DAD, including protocatechuic acid, vanillic acid, flavonoids, ellagic acid, quercetin 3-glucoside, quercetin, kaempferol, and isorhamnetin [146]. Furthermore, the ethyl acetate fraction obtained from the outer layers of the bulb showed activity against H295R adrenal cancer cells, with 41.39% inhibition at 30 µg/mL, superior to the mitotane positive control (33.47%). This fraction exhibited high concentrations of free quercetin and quercetin monoglucosides, suggesting that these flavonoids play a central role in the observed activity. Furthermore, cell cycle arrest was observed in G1 and G2 phases, indicating direct interference with cell proliferation (determined by 7-aminoactinomycin D staining assays and measurement of apoptosis and necrosis rates by flow cytometry) [147].

However, the use of nanoparticles, such as silver or chitosan, significantly increases their efficacy, achieving lower IC_50_ values in various cell models. Silver nanoparticles synthesized from the peel extract showed activity against A549 cells (IC_50_ of 113.25 µg/mL at 24 h). The mechanism of action was related to the induction of apoptosis, evidenced by Hoechst 33258 and AO/EB staining, as well as alterations in mitochondrial membrane potential. These results suggest activation of the intrinsic apoptotic pathway associated with mitochondrial damage [142].

Silver nanoparticles synthesized from leaves exhibited inhibition of MCF-7 cells, reaching 86% inhibition at 200 µg/mL. The proposed mechanism involved direct interaction of the nanoparticles with intracellular proteins, DNA, and phosphate groups, as well as the generation of reactive oxygen species that cause cellular oxidative damage (determined by a DPPH test) [143]. One of the most relevant results corresponded to the onion extract encapsulated in chitosan nanoparticles, which showed a marked improvement in cytotoxic activity against various tumor cell lines. IC_50_ values ranged from 10.29 to 35.15 µg/mL, showing considerably higher activity than the extract alone (between 33.29 and 96.39 µg/mL). The mechanism involved a significant increase in caspases-3 and -9 and a decrease in BCL-2, confirming the induction of mitochondrial apoptosis (determined by ELISA). Phytochemical analysis indicated a high content of flavonoids, phenols, tannins, alkaloids, saponins and steroids [145].

### 4.2. Garlic

Garlic (*Allium sativum* L.) has an annual global production of 28 million tons across approximately 1.6 million hectares, with China and India being the largest producers, accounting for 80% of world production [148]. Garlic is used as a flavoring and is one of the most cultivated and studied functional plants among the lilies [149,150]. It has long been considered a medicinal food and is important in the prevention of various diseases, such as cancer [151,152,153]. Table 8 shows recent studies on garlic as an antiproliferative agent against cancer cells.

Table 8 shows that garlic exhibits significant in vitro anticancer activity, positioning garlic as one of the species with the greatest antiproliferative effect among the evaluated plants. The hydroalcoholic extract of garlic bulbs showed high cytotoxicity against DLD-1, MDA-MB-231, MCF-7, and SK-MES-1 cells, with IC_50_ values between 4.65 and 6.37 µg/mL. However, although potency was notable, selectivity was limited because normal human fibroblasts (BJ) and keratinocytes (HaCaT) also showed relatively high sensitivity (IC_50_ = 8.84 and 10.86 µg/mL, respectively). The mechanism of action was primarily associated with necrosis, determined by Annexin V/PI flow cytometry, suggesting severe cell damage rather than programmed apoptosis. Phytochemical analysis identified allicin, alliin, chlorogenic acid, *p*-coumaric acid, and 4-hydroxybenzoic acid using HPLC-DAD-MS. Among these metabolites, allicin stands out for its recognized ability to induce oxidative stress, alter mitochondrial metabolism, and modulate apoptotic and antiproliferative pathways [154].

Furthermore, the use of extracellular nanovesicles and metallic nanoparticles synthesized from garlic extracts appears to enhance or diversify their biological activity, highlighting their potential as a platform for biotechnological applications. Garlic-derived extracellular nanovesicles showed significant activity against A498 and A549 cells, achieving inhibitions of 78% and 72%, respectively. The mechanism involved apoptosis and decreased VEGF expression, further suggesting an anti-angiogenic effect (determined by annexin V/PI staining and analysis of apoptotic mRNA and protein expression levels). However, the inhibition observed in normal dermal fibroblasts (~60%) again indicates limited selectivity [155]. Gold nanoparticles synthesized from aqueous extracts of branches and leaves significantly improved cytotoxic activity compared to the extract alone. IC_50_ values obtained against HT-29, HTC116, HCT-8, and Ramos.2G.4C10 ranged from 225 to 269 µg/mL, while chloroauric acid showed low inhibition percentages (<25% at 125 µg/mL), and the garlic extract alone showed IC_50_ values greater than 360 µg/mL. Furthermore, the nanoparticles showed low toxicity in HUVEC cells (~12% inhibition at 1000 µg/mL), indicating more favorable biological selectivity [156].

The stem extract showed a moderate effect against murine melanoma B16-F0, associated with downregulation of VEGF, MMP-2, and MMP-9 [determined by RT-PCR], suggesting potential antiangiogenic and antimetastatic activity. Importantly, it showed no toxicity in normal renal cells (HEK-293), demonstrating favorable selectivity. HPLC analysis identified gallic acid as one of the relevant metabolites [157]. While silver nanoparticles synthesized from ethanolic extract significantly increased activity against A549 (IC_50_ = 22 µg/mL), compared to the extract alone (IC_50_ = 177 µg/mL), again demonstrating the enhancing effect of nanoparticles on anticancer activity.

### 4.3. Leek

The leek (*Allium ampeloprasum*) is an edible vegetable with a white neck and dark green leaves [163,164,165]. As one of the world’s most important vegetable crops, particularly in Western Europe, according to data from the Food and Agriculture Organization of the United Nations, leeks are produced worldwide, with an approximate production of 2,179,050 tons and a cultivated area of 137,791 hectares in 2017 [166]. Studies have described some antioxidants, antibacterial, anti-inflammatory, antidiabetic, antihypertensive, antifungal, and antiproliferative properties [163,164,167,168]. Although few studies have been conducted, Table 8 presents some of the research that has been carried out, primarily on breast and lung cancer. Table 8 shows that leek exhibits variable but promising antiproliferative activity against different tumor cell lines. In MCF-7 cells, both the aqueous and methanolic extracts showed moderate inhibition of cell growth (52.84 and 40.86% inhibition at 50 μg/mL, respectively). UPLC-QTOF/MS analysis identified metabolites such as entadamide A-β-D-glucopyranoside, xantiside, entanamide A, E-ajoene, and xantiazone [159]. More pronounced effects were observed in liver cancer models, particularly with the methanolic leaf extract (IC_50_ of 38.47 µg/mL), comparable to the effect of metformin (IC_50_ of 38.99 µg/mL), indicating that biological efficacy depends on both the type of extract and the tumor model evaluated. Table 8 also shows that the methanolic leaf extract was associated with increased expression of the P53 gene (determined by real-time PCR analysis), suggesting a possible effect on tumor suppression pathways and cell cycle control. Furthermore, the combination of leaf extract with metformin resulted in a marked reduction in the IC_50_ value to 1.36 µg/mL, indicating a possible synergistic effect (which would have to be verified by a quantitative synergy model) and reinforcing the interest in leek as an adjuvant in therapeutic strategies [160]. Although some nanostructured formulations, such as silver and gold nanoparticles, also showed activity, their potency was more moderate or dependent on the cell type. Silver nanoparticles synthesized from aqueous leaf extract showed activity against several tumor cell lines, with IC_50_ values between 125 and 180 µg/mL. However, they showed no cytotoxic effect on normal HUVEC cells, indicating a favorable selectivity for tumor cells [161]. In contrast, gold nanoparticles obtained from aqueous extract showed low activity against MDA-MB-231 (IC_50_ = 483.9 µg/mL) [162]. Overall, the results position leek as a species with significant antiproliferative potential, although further studies are needed to more precisely define its mechanisms of action and biological applicability.

### 4.4. Chives

Chives (*Allium schoenoprasum* L.), commonly known as scallions, have great culinary value in addition to their use in ethnomedicine [169]. Although the literature on *A. schoenoprasum* L. is scarce, scientific research on chives validates traditional claims and demonstrates potential anticancer pharmacological properties [169].

Among these studies is the one conducted by Hsing et al. [170], who investigated the association between the incidence of prostate cancer and the consumption of vegetables of the genus *Allium* (chives, garlic, onion, spring onion, and leek) in men with confirmed prostate cancer from the Chinese population. They observed that men who consumed more than 10 g of vegetables of the genus *Allium* (including chives) per day had a significantly lower risk of cancer than those who consumed less than 2.2 g/day. The reduction in prostate cancer risk associated with these vegetables was independent of body size, intake of other foods, and total caloric intake, and was more pronounced in men with localized prostate cancer than in men with advanced prostate cancer.

Similarly, Setiawan et al. [171] and Zhou et al. [172] examined the association between the consumption of these vegetables and cancer. Furthermore, a meta-analysis reported that the consumption of vegetables from the genus *Allium* (including chives) reduced the risk of stomach and gastric cancer in the Chinese population, reinforcing the possibility that chives may possess anticancer activity. However, they did not confirm whether the reduced risk of prostate, stomach, or gastric cancer was directly associated with chive consumption. Therefore, further studies are needed to establish the anticancer properties of chives.

### 4.5. Summary and General Perspectives

The anticancer activity observed in species of the Liliaceae family, particularly onions, demonstrates that solvent polarity significantly influences the recovery of bioactive compounds and, consequently, the biological efficacy of the resulting extracts. Extracts obtained using solvents of intermediate polarity, such as ethanol and ethyl acetate, showed a greater capacity to recover flavonoids and phenolic compounds associated with cytotoxic activity, including quercetin, kaempferol, protocatechuic acid, vanillic acid, and isorhamnetin. On the other hand, aqueous or less selective extracts tend to recover highly polar compounds with predominantly antioxidant activity, but with lower direct cytotoxic potency. This relationship between polarity and bioactivity coincides with the trend observed in other medicinal plants, where hydroalcoholic extracts or flavonoid-enriched fractions exhibit greater antiproliferative activity than conventional aqueous extracts.

Although several studies have described mechanisms associated with apoptosis, oxidative stress, and other factors, some work still lacks elucidation of the mechanisms of action. In this regard, future research should incorporate transcriptomic and proteomic analyses, among others, to identify specific molecular pathways modulated by plant extracts. The implementation of omics analyses would also allow for the clarification of synergistic effects between metabolites present in complex extracts, since numerous studies suggest that biological activity does not depend exclusively on a single compound, but rather on the interaction between flavonoids, sulfur compounds, phenols, and antioxidants. In the case of onion, the high presence of quercetin and its derivatives could act in conjunction with organosulfur compounds to enhance pro-apoptotic and anti-proliferative mechanisms.

The use of plant nanoparticles also represents a strategy to improve targeted drug delivery and overcome tumor resistance mechanisms. Among the species evaluated, onion showed particularly consistent activity, especially when the extracts were nanoencapsulated as metallic nanoparticles. The IC_50_ values obtained with chitosan nanoparticles (10.29–35.15 µg/mL) showed considerably greater activity than conventional extracts, demonstrating that nanotechnology strategies significantly improve biological efficacy.

From a nutraceutical perspective, the results support the dietary consumption of vegetables from the Liliaceae family as part of cancer prevention strategies. Compounds such as quercetin, kaempferol, isorhamnetin, and phenolic acids possess recognized antioxidant, anti-inflammatory, and chemopreventive properties. The regular inclusion of onion, garlic, and other members of the Allium genus in the diet could contribute to reducing oxidative damage and modulating inflammatory processes related to carcinogenesis. In particular, the outer layers of onions, frequently discarded as agro-industrial waste, represent a concentrated source of bioactive flavonoids with potential for the development of nutraceutical supplements or bioactive ingredients for pharmaceutical formulations.

## 5. Chenopodiaceae Family

The Chenopodiaceae family is currently classified within the Chenopodioideae subfamily of the Amaranthaceae family. This family comprises approximately 110 genera and over 1700 species [173]. It has a wide geographic distribution and is found in various regions of the world, including North America, the Mediterranean coast, the Red Sea coast, Australia, and Central Asia. The most consumed vegetables are chard, spinach, and beets [174,175]. Plants of the Chenopodiaceae family have been valued for their medicinal properties in different cultures around the world. Its traditional uses include the treatment of various conditions and diseases, such as digestive problems, inflammation, infections, joint pain, diabetes, and cancer [175,176,177].

### 5.1. Beetroot

Beetroot (*Beta vulgaris* L.) is a vegetable of great commercial and nutritional importance. Its root is reddish purple and is consumed in salads, cooked, or juiced [178,179]. Western Europe is the largest producer of beetroot, with a yield of around 130 tons per hectare [180]. Beetroot extracts have been described as potential chemopreventive agents that decrease cell proliferation and induce apoptosis [181,182].

In this regard, Piasna-Słupecka et al. [183] prepared and subjected to in vitro gastrointestinal digestion and absorption of juices from fourteen-day-old and fully mature beetroot shoots and roots to treat the MCF-7 and MDA-MB-231 cell lines. The results showed that the addition of digested juice from young shoots to the MCF-7 cell line significantly inhibited cell proliferation by 27.38% after 24 h, and by 33.0% and 58.83% at 48 and 72 h, respectively. On the other hand, the addition of digested root juice to the MCF-7 cell line reduced cell proliferation by 22.9, 30.59, and 63.46% after 24, 48, and 72 h, respectively. Similarly, digested juice from young shoots inhibited proliferation by 4.76, 9.11, and 13.19% after 24, 48, and 72 h, respectively, in MDA-MB-231 cells. Juice subjected to in vitro digestion and gastrointestinal absorption significantly inhibited cell proliferation in the MDA-MB-231 cancer cell line after 72 h of incubation, by almost 10%.

Furthermore, evidence has been provided of the antiproliferative effect of different types of beet extracts and nanoparticles against various cancer cells (Table 9).

Table 9 shows that the analyzed beetroot studies exhibit variable anticancer activity depending on the type of extract, the extraction method, and the nanoparticle used. The ethanolic root extract showed moderate inhibition of growth in A549 cells (22% at 800 µg/mL), suggesting low cytotoxic potency compared to other beetroot derivatives. However, this extract contained various phenolic compounds and flavonoids, including gallic acid, ferulic acid, myricetin, kaempferol, and apigenin (determined by HPLC analysis). The low activity observed could be associated with insufficient concentrations of these bioactive compounds or with possible limitations in their cellular bioavailability [184]. The hydroalcoholic root extract showed more significant activity in Caco-2 and HT-29 cell lines, with IC_50_ values close to 100 µg/mL, without exhibiting toxicity to normal cells (KDR/293) even at 140 µg/mL. It also displayed a pro-apoptotic mechanism characterized by upregulation of BAD, Fas-R, and caspases, along with a decrease in Bcl-2 (as determined by DAPI staining, FACS flow cytometry, and RT-PCR). Analysis of purified betanin revealed greater potency (IC_50_ values of 90 and 64 µg/mL, respectively) than the crude extract, suggesting that this pigment is one of the main compounds responsible for the observed bioactivity [187]. However, some preparations, such as flour or fermented products, show lower efficacy against MCF-7 and MDA-MB-231 cells, with LC_50_ values in the mg/mL range, indicating low cytotoxic potency. Nevertheless, metabolomic characterization revealed a diversity of betalains and flavonoids, including betanin, vulgaxanthins, and feruloylated derivatives, compounds associated with antioxidant capacity and chemopreventive potential [186].

In contrast, beetroot fermented with water kefir produced a 77.72% inhibition of HepG2, associated with apoptosis (determined by an annexin V-FITC and PI assay) and enrichment in specific betalains such as betanidin, neobetanin, and decarboxylated derivatives (determined by LC-MS). Correlation analysis showed that these metabolites were positively associated with anticancer activity, demonstrating that fermentation processes can favorably modify the phytochemical profile and enhance the bioactivity of plant extracts [188]. Furthermore, the use of nanoparticles derived from the extract, such as copper oxide, significantly improves cytotoxic activity, achieving lower IC_50_ values of 25 µg/mL in A549 cells. Regarding the mechanisms of action, a trend toward the induction of apoptosis is observed, accompanied by the upregulation of pro-apoptotic genes (such as caspases and BAD), a decrease in anti-apoptotic proteins (Bcl-2), the generation of reactive oxygen species, and cell cycle arrest, particularly in the G2/M phase [185].

### 5.2. Spinach

Spinach (*Spinacia oleracea* L.) is one of the most consumed and nutritious vegetables worldwide [196]. In 2016, global spinach production was approximately 27 million tons, with China being the largest producer [196]. In addition to being a proven source of essential nutrients, spinach has also been described as having antiproliferative effects in different cancer cell lines [197,198,199,200]. In this regard, Table 9 shows the in vitro biological effects of different spinach extracts against colorectal, cervical, and leukemia cancer cell lines. The hydroalcoholic extract exhibited approximately 80% inhibition of HT-29 cells at 500 µM, associated with increases in intracellular reactive oxygen species (determined by comet assays). This suggests that the mechanism of action may involve induced oxidative stress, leading to cell damage and tumor death [189]. Leaf extracts showed moderate inhibition under normoxic conditions (59.56 This increase was correlated with significant changes in the phytochemical profile, with higher concentrations of ascorbic acid, ferulic acid, α-tocopherol, and various glucuronidated flavonoids detected by LC-MS/MS. These results indicate that hypoxic stress stimulates the accumulation of antioxidant and phenolic metabolites capable of enhancing the plant’s biological activity (verified by HPLC-MS/MS analysis and the aluminum chloride and ABTS assays) [190].

On the other hand, the 75% ethanolic extracts demonstrated high inhibition (up to 88.9%) in the K562 cell line, comparable even to positive controls such as doxorubicin and taxol (86% and 79%, respectively) under the evaluated conditions, showing a more pronounced response in certain cell types [194]. Furthermore, the 80% ethanolic extract showed potent cytotoxic activity in HeLa cells (IC_50_ = 13.80 µg/mL), associated with the induction of apoptosis determined by DAPI, annexin V, and PI staining, demonstrating activation of programmed cell death [192]. The difference in activity between extracts is likely reflected in variations in phytochemical composition related to the solvent and extraction conditions.

### 5.3. Chard

Swiss chard (*Beta vulgaris* L. var. *cicla o flavescens*) is a leafy green vegetable cultivated globally not only for its culinary versatility but also for its nutritional profile [201,202]. Swiss chard extracts have been studied due to their positive effects on human health [201,203,204]. These include their protective effects against oxidative stress and chronic diseases, such as cardiovascular disease and cancer [202,205].

Some of the studies on Swiss chard are those presented in Table 9, which. shows that chard exhibits anticancer activity, with cytotoxic effects in different cell lines. Among these, the phenolic fraction of the leaves showed high potency in MCF-7 cells, with an IC_50_ of 9.1 µg/mL, associated with inhibition of DNA synthesis (determined by a [3*H*]thymidine incorporation assay). Rhamnosylated vitexin, xylosylvitexin, isorhamnetin gentiobioside, and rutin were identified as predominant metabolites [193]. Furthermore, the ethyl acetate extract from the seeds showed activity in RKO cells with an IC_50_ of 32 µg/mL, inducing apoptosis and alterations in the cell cycle, particularly an increase in the G1 phase and a decrease in the S phase (determined by flow cytometry). A particularly relevant aspect was the increased proliferation observed in normal human fibroblasts, which could indicate a degree of tumor selectivity and low toxicity to healthy cells. The metabolites identified in this fraction included phenolic aldehydes, vanillic acid, and glycosylated flavonoids derived from rhamnetin (identified by HPLC-ESI-MS) [194].

On the other hand, the extracts obtained under different agronomic conditions (with and without irrigation and fertilization treatment) showed similar IC_50_ values in MCF-7 cells (20.76 and 23.33 µg/mL, respectively), indicating that these variables can influence, although not drastically, their biological activity. The main metabolites identified were vitexin and isorhamnetin derivatives, flavonoids associated with antioxidant and antiproliferative properties (determined by ESI MS conditions and UV-vis spectra). Studies conducted with chard showed significant cytotoxic activities, particularly associated with phenolic fractions enriched in glycosylated flavonoids.

### 5.4. Summary and General Perspectives

In species of the Chenopodiaceae family, the observed anticancer activity was influenced by the nature of the solvent used during extraction, demonstrating that solvent polarity plays a fundamental role in the recovery of bioactive compounds. In general, hydroalcoholic and ethanolic extracts showed greater antiproliferative activity compared to aqueous extracts, probably due to their ability to simultaneously solubilize metabolites of intermediate polarity such as flavonoids, betalains, phenolic acids, and glycosylated derivatives. For example, hydroalcoholic extracts of chard showed IC_50_ values close to 100 µg/mL in Caco-2 and HT-29 cell lines, associated with the upregulation of pro-apoptotic genes and a decrease in Bcl-2, while aqueous extracts of bran flour showed lower cytotoxic potency, with LC_50_ values in the mg/mL range. This suggests that more polar solvents favor the extraction of hydrophilic compounds, although not necessarily of the metabolites with the greatest anticancer activity.

Similarly, in spinach, 75% and 80% ethanolic extracts showed activity against K562 and HeLa cells, indicating that intermediate ethanol concentrations allow for more efficient extraction of flavonoids, tocopherols, and phenolic compounds with pro-apoptotic capacity. Furthermore, the increase in antioxidant metabolites under hypoxic conditions demonstrated that not only the solvent but also the plant’s physiological stress modifies the phytochemical profile. In the case of chard, the phenolic fractions and ethyl acetate extracts exhibited particularly potent activities, demonstrating that solvents of intermediate polarity favor the recovery of glycosylated flavonoids and phenolic aldehydes associated with the inhibition of DNA synthesis and alterations in the cell cycle. The results also show that certain species exhibited more consistent and potent anticancer activity. The phenolic fraction of chard leaves stood out for having the lowest IC_50_ values (IC_50_ = 9.1 µg/mL in MCF-7), suggesting a high concentration of bioactive flavonoids such as vitexin, rutin, and isorhamnetin. Similarly, ethanolic extracts of spinach showed remarkable activity against HeLa and K562 cells, comparable even to reference chemotherapeutic agents under certain experimental conditions. Furthermore, beetroot-derived nanoparticles also exhibited significant activity, particularly copper oxide nanoparticles, which reached IC_50_ values of 25 µg/mL, demonstrating that nanoparticles can enhance the bioavailability and activity of plant metabolites.

Finally, many of these results support the nutraceutical potential of Chenopodiaceae species due to their abundance of betalains, flavonoids, and bioactive phenolic compounds. Metabolites such as betanin, neobetanin, vitexin, rutin, ferulic acid, and α-tocopherol have been associated with antioxidant, anti-inflammatory, and chemopreventive properties. In this context, regular consumption of beets, spinach, and chard could contribute to reducing oxidative stress and modulating processes related to carcinogenesis. Therefore, it is recommended to promote dietary patterns rich in minimally processed fresh vegetables, functional juices, and fermented foods derived from these species, since processes such as fermentation can increase the bioavailability and biological activity of specific compounds. Furthermore, incorporating these vegetables into balanced Mediterranean-style or plant-based diets could support complementary preventive strategies against chronic diseases and cancer.

## 6. Conclusions

In this review, the results reveal that several edible plants belonging to the Cucurbitaceae, Brassicaceae, Liliaceae, and Chenopodiaceae families possess remarkable in vitro antiproliferative activity. Extracts, fractions, and nanoparticles derived from these foods exhibit effects in multiple tumor cell lines, primarily associated with the induction of apoptosis, cell cycle arrest, modulation of signaling pathways, and regulation of oxidative stress. Although the results are promising, further in vitro assays on the mechanism of action and in vivo studies are needed to validate their efficacy and safety. Collectively, these plants represent a relevant source for the development of new nutraceutical and complementary therapeutic strategies in cancer treatment.

## Figures and Tables

**Table 1:** Distribution of selected foods according to their botanical family.

Family	Food
Cucurbits	Pumpkin, squash, zucchini, cucumber, watermelon, melon
Brassicas	Broccoli, cauliflower, watercress, radish, cabbage, arugula, Brussels sprouts, kale
Liliaceae	Garlic, onion, leek, chives
Chenopodiaceae	Swiss chard, spinach, beetroot

**Table 2:** In vitro anticancer activity and associated mechanisms of extracts and nanoparticles derived from *Cucurbita* spp.

Product Used	Cell Line	In Vitro Activity	Mechanism of Action	Reference
Pumpkin (*Cucurbita* spp.)				
Hydroalcoholic extract of leaves (*C. pepo*)	HepG2	IC_50_ = 132.6 µg/mL	ND	[27]
	CT26	IC_50_ = 167.2 µg/mL		
Hydroethanolic extract of seeds (*Cucurbita pepo* L. subsp. *pepo* var styriaca)	Du-145 (Prostate carcinoma)	Inhibition = ~ 40–50%	ND	[28]
	LnCaP (Metastatic prostate adenocarcinoma)			
	BPH-1 (Benign prostatic hyperplasia)			
	Caco-2			
	MCF-7			
Hydroalcoholic extract of hulless seed (*Cucurbita* spp.)	Papillary thyroid cancer (unterminated cell line)	IC_50_ = 1.31 µg/mL at 24 h	ND	[29]
Seed oil extract (*C. maxima* L. cv. Nychaki)	HeLa	GI_50_ = 270 µg/mL	ND	[30]
Silver nanoparticles from hydroethanolic extract fruit (*C. pepo* L.)	MCF-7	GI_50_ = 34.67 µg/mL at 50 µg/mL	Apoptosis	[31]
Copper oxide nanoparticles from seed extract (*Cucurbita* spp.)	MDA-MB-231	IC_50_ = 20 µg/mL	Morphological changes such as contraction, detachment, blistering of the membrane, and distortion of shape and apoptosis	[32]
Zinc oxide nanoparticles from seed extract (*Cucurbita* spp.)	MDA-MB-231	IC_50_ = 10 µg/mL	Inhibition of cell adhesion and migration, and apoptosis	[33]
Cu-Mn nanoparticles from seed extract (*Cucurbita* spp.)	HT-29	IC_50_ = 115.2 µg/mL	significant reduction in cell migration	[34]
Aqueous extract of seeds (*C. máxima*)	MCF-7	IC_50_ = 45.40 µg/mL at 24 h	Apoptosis	[35]
Extract (*C. pepo*)	A549	IC_50_ = 33 µg/mL at 24 h	ND	[36]
Chitosan nanoparticles loaded with seed oil (*C. pepo*)	SCC-25 (carcinoma)	IC_50_ = 22 µg/mL at 48 h	Significant increase in caspase 9 expression	[37]
Silver nanoparticles from peel extract (*Cucurbita* spp.)	MDA-MB-231	IC_50_ = 4.3 µg/mL	Apoptosis, radiation-induced inhibition of HIF-1α, and decreased expression of cyclin D1 and p-ERK	[38]
Silver nanoparticles from peel extract (*Cucurbita* spp.) in combination with radiotherapy at 8 Gy		Combination index (IC) = 0.49 at 5 µM at 48 h (IC < 1 synergistic effect)	Increased overexpression of genes related to apoptosis	
Silver nanoparticles from seeds (*C. pepo* L.)	HCT-116	Inhibition = 40% at 1000 µg/mL at 24 h	ND	[39]
Aqueous extract of seeds (*Cucurbita* spp.)	HCT-116	IC_50_ = 213.59 µg/mL	ND	[40]
	A549	IC_50_ = 208.72 µg/mL		
Seed extract (*C. pepo*)	MEC-1 (mutant p53 chronic B cell leukaemia) HG-3 (Chronic B-cell leukemia)	IC_50_ = 205 µg/mL at 24 h IC_50_ = 209 µg/mL at 24 h	ND	[41]
Silver nanoparticles from aqueous leaf extract (*C. máxima*)	SiHa (Cervical cancer)	IC_50_ = ~8 µg/mL	ND	[42]

ND: Not determined.

**Table 3:** In vitro anticancer activity of extracts, oil, and nanoparticles derived from cucumber and watermelon.

Product Used	Cell Line	In Vitro Activity	Mechanism of Action	Reference
**Cucumber (*Cucumis sativus* L.)**				
Silver nanoparticles from plant extract	Pa-1 (Ovarian teratocarcinoma)	Inhibition = 49.51% at 50 µg/mL	Apoptosis	[49]
		IC_50_ = 49.71 µg/mL		
Seed oil (*C. sativus*)	DU145	Inhibition = ~70% at 100 µg/mL at 48 h	Increased number of apoptotic cells and inhibition of cancer cell migration and invasion, while decreased adhesion to the extracellular matrix of collagen and fibrinogen	[12]
Seed oil (*C. maxima*)	DU145	Inhibition = ~70% at 100 µg/mL at 48 h	Decreased levels of IL-1β, IL-6 and TNFα	[50]
	PC3 (Prostate cancer)			
Foamy cucumber extract	MDA-MB-231	IC_50_ = 116.11 µg/mL	Induction of apoptosis	[45]
Wild cucumber extract (*C. pubescens*)	A549	Inhibition = 77% at 100 µg/mL	ND	[51]
		IC_50_ = 7.5 µg/mL at 24 h		
**Watermelon (*C. lanatus*)**				
Nanoparticles from seed extract	HCT-116	IC_50_ = 33.7 µL at 100 µL	ND	[52]
	HepG2	IC_50_ = 44.0 µL at 100 µL		
	HeLa	IC_50_ = 70.6 µL at 100 µL		
Rind aqueous extract	HCT-116	IC_50_ = 24 µg/mL	It triggered apoptosis and boosted the accumulation of cells in the S phase, raising caspase-3 activity and the BAX/BCL-2 ratio	[53]
	HepG2	IC_50_ = 20 µg/mL		
Extracts from the rind	HRAC-769-P (Adenocarcinoma)	Inhibition = 66% at 156.8 mg mL^−1^ at 24 h	Early polycaspase response and a significant reduction in cell migration. In addition, expression of genes associated with apoptosis, such as BMF, NPTX1, NFKBIA, NFKBIE, and NFKBID, which could induce intrinsic and extrinsic apoptosis	[54]
Ethanolic extract of the seeds	A549	IC_50_ = 51.73 µg/mL	Induced cell cycle arrest and apoptosis in the G2 phase	[55]

ND: Not determined.

**Table 4:** In vitro anticancer activity of extracts, juice, and nanoparticles derived from radish and kale.

Product Used	Cell Line	In Vitro Activity	Mechanism of Action	Reference
**Radish (*Raphanus sativus* L.)**				
Hexane root extract	HeLa	IC_50_ = 8.78 μg/mL at 24 h	Apoptosis and interactions between genes of the Bcl(2) family, overexpression of pro-apoptotic genes and underexpression of anti-apoptotic genes	[78]
		IC_50_ = 7.40 μg/mL at 48 h		
		IC_50_ = 7.15 μg/mL at 72 h		
	A549	IC_50_ = 10.24 μg/mL at 24 h		
		IC_50_ = 8.03 μg/mL at 48 h		
		IC_50_ = 7.71 μg/mL at 72 h		
	MCF-7	IC_50_ = 8.36 μg/mL at 24 h		
		IC_50_ = 7.64 μg/mL at 48 h		
		IC_50_ = 7.51 μg/mL at 72 h		
	PC-3	IC_50_ = 20.87 μg/mL at 24 h		
		IC_50_ = 14.92 μg/mL at 48 h		
		IC_50_ = 12.96 μg/mL at 72 h		
Ethanolic extract of leaves	MDA-MB-231	Significant inhibition at 48 h	Inhibition via the ErbB-Akt pathway	[79]
Root extract	MCF-7	IC_50_ = 306.3 μg/mL	ND	[80]
	MDA-MB-231	IC_50_ = 470.0 μg/mL		
	HepG2	IC_50_ = 444.6 μg/mL		
	A549	IC_50_ = 250.6 μg/mL		
Leaf extract	MCF-7	IC_50_ = 217.0 μg/mL		
	MDA-MB-231	IC_50_ = 287.0 μg/mL		
	HepG2	IC_50_ = 224.0 μg/mL		
	A549	IC_50_ = 453.2 μg/mL		
Graphene oxide reduced from white radish extract	A549	IC_50_ = 26.69 μg/mL	ND	[81]
	MCF-7	IC_50_ = 33.22 μg/mL		
Leaf extract	MCF-7	IC_50_ = 95.43 μg/mL	Apoptosis	[82]
Zinc oxide nanoparticles from leaf extract	MCF-7	IC_50_ = 8.05 μg/mL		
Silver nanoparticles from leaf extract	Caco-2	Inhibition = 56% at 100 μg/mL	ND	[83]
	SK-OV-3	Inhibition = 50% at 100 μg/mL		
	U118-MG	Inhibition = 20% at 100 μg/mL		
	HDF (Dermal fibroblasts)	Inhibition = 33% at 100 μg/mL		
Zinc oxide nanoparticles from root	A549	IC_50_ = 22.79 μg/mL	Reduction in inflammasome activity by inducing autophagy	[84]
	MCF-10 (normal breast cell)	IC_50_ = 272.24 μg/mL		
**Kale (*Brassica oleracea* var.)**				
Aqueous extract	A549 (Lung cancer)	Inhibition = 41% at 500 μg mL^−1^	ND	[85]
Ethanolic extract	PC-3	IC_50_ = 351.7 μg/mL	Apoptosis, increased mRNA and protein of the NRF2 and BAX pathway genes, decreased genes of the NF-κB pathway, BCL-2 and MMP	[86]
Leaf extract	HeLa (Cervical cancer)	Inhibition = 23% at 50 µg mL^−1^	ND	[87]
Fresh juice	U937 (Myelomonocytic leukemia)	Inhibition = 87%	Apoptosis and increased the amount of activated caspase-3, while the amounts of Bcl-2 and Bax were not modified	[88]
Steamed juice		Inhibition = 51%		
Methanolic Extract (Vates Blue Curled)	HepG2 (Liver cancer)	Inhibition = 78.67% a 100 µg/mL	ND	[89]
Methanolic extract (Red Russian)		Inhibition = 70.07% at 100 µg/mL		
Methanolic extract (Lacinato)		Inhibition = 56.85% at 100 µg/mL		
Methanolic extract (Prumier)		Inhibition = 64.73% at 100 µg/mL		
Methanolic extract of sprouts treated with γ-polyglutamic acid	HCT116	IC_50_ = 130.1–153.4 μg/mL	ND	[90]

ND: Not determined.

**Table 6:** In vitro anticancer activity of extracts, oil, and nanoparticles derived from Brussels sprouts, cabbage, and cauliflower.

Product Used	Cell Line	In Vitro Activity	Mechanism of Action	Reference
**Brussels sprouts (*Brassica oleracea* var. *gemmifera*)**				
Aqueous extract	A549	Inhibition = 28% at 500 μg/mL at 72 h	ND	[85]
Ethanolic extract	MDA-MB-231	IC_50_ = 210.41 µg/mL at 24 h	ND	[123]
**Cabbage (*Brassica oleracea* var. *capitata*)**				
Aqueous extract	A549	Inhibition = 63% at 500 μg mL^−1^ at 72 h IC_50_ = 38 μg/mL	ND	[85]
Cabbage extract	HeLa	IC_50_ = 23.38 µg/mL	Apoptosis, increased levels of TNFα, arrest of the G0/G1 phase of the cell cycle	[124]
	HepG2	IC_50_ = 28.66 µg/mL		
Juice of young shoots	DU145	Inhibition = 46% at 72 h	ND	[125]
	LNCap (Prostate cancer)	Inhibition = 40% at 72 h		
Juice mature vegetable	DU145	Inhibition = 30% at 72 h		
	LNCap	Inhibition = 29% at 72 h		
Alcoholic extract	HeLa	IC_50_ = 22.78 µg/mL	ND	[126]
	MCF-7	IC_50_ = 47.84 µg/mL		
	HepG-2	IC_50_ = 69.11 µg/mL		
Phenolic extract	HeLa	IC_50_ = 17.71 µg/mL		
	MCF-7	IC_50_ = 28.89 µg/mL		
	HepG-2	IC_50_ = 21.08 µg/mL		
**Cauliflower (*Brassica oleracea* var. *botrytis*)**				
Aqueous extract of purple cauliflower	HT29	Inhibition = 75% at 24 h	ND	[127]
Aqueous extract of white cauliflower		Inhibition = 25% at 24 h		

ND: Not determined.

**Table 7:** In vitro anticancer activity of onion extracts and nanoparticles and their mechanism of action have been reported.

Product Used	Cell Line	In Vitro Activity	Mechanism of Action	Reference
	Onion (*Allium* cepa L.)			
Silver nanoparticles from peel extract	A549	IC_50_ = 113.25 µg/mL at 24 h	Apoptosis	[142]
Silver nanoparticles from plant leaves	MCF-7	Inhibition = 86% at 200 µg/mL	Effective interaction of nanoparticles with functional groups of intracellular proteins, nitrogenous bases, and phosphate groups in DNA. Excitation of immediate oxygen species and damage to cellular components	[143]
Peel extract	HT-29	Inhibition = 62.93% at 100 µg/mL	Apoptosis through negative regulation of the expression of L1 cell adhesion molecule (L1CAM) signaling pathways and inhibition of cell migration and invasion	[144]
Onion extract encapsulated in chitosan nanoparticles	AsPC-1 (Pancreatic cancer)	IC_50_ = 35.15 µg/mL	nduction of apoptosis by s ignificantly increasing in caspase-3 and -9 activity, and decreasing BCL-2 concentration	[145]
	MCF-7	IC_50_ = 10.29 µg/mL		
	HCT116	IC_50_ = 12.43 µg/mL		
	Hep2 (Squamous cell carcinoma)	IC_50_ = 18.32 µg/mL		
	HepG2	IC_50_ = 14.90 µg/mL		
Onion extract	Caco-2	Inhibition = ~55% at 1000 µg/mL at 48 h	Alteration of the onset of the cell cycle, showing arrest in the S phase with a decrease in the G1 phase and an increase in the number of cells that had the P53 protein active and caspase 3 activation	[146]
Ethyl acetate fraction of the outer layer of onion bulbs	H295R (Adrenal cancer)	Inhibition = 41.39% at 30 µg/mL at 48 h	Increase in G2 phase and arrest of G1 phase of the cell cycle	[147]

ND: Not determined.

**Table 8:** In vitro anticancer activity of extracts and nanoparticles derived from garlic and leek.

Product Used	Cell Line	In Vitro Activity	Mechanism of Action	Reference
**Garlic (*Allium sativum* L.)**				
Hydroalcoholic extract of garlic bulbs	DLD-1 (Colorectal cancer)	IC_50_ = 5.48 µg/mL	Necrosis	[154]
	MDA-MB-231	IC_50_ = 6.37 µg/mL		
	MCF-7	IC_50_ = 6.13 µg/mL		
	SK-MES-1 (Lung cancer)	IC_50_ = 4.65 µg/mL		
Extracellular nanovesicles	A498 (Kidney cancer)	Inhibition = 78% at 50 μg/mL at 72 h	Apoptosis, decreased expression of the angiogenic VEGF protein	[155]
	A549	Inhibition = 72% at 50 μg/mL at 72 h		
Gold nanoparticles from aqueous extracts of branches and leaves	HT-29	IC_50_ = 269 µg/mL at 48 h	ND	[156]
	HTC116	IC_50_ = 225 µg/mL at 48 h		
	HCT-8 (Colorectal cancer)	IC_50_ = 250 µg/mL at 48 h		
	Ramos.2G.4C10 (Burkitt lymphoma)	IC_50_ = 236 µg/mL at 48 h		
Stem extract	B16-F0 (Murine melanoma)	Inhibition = 30.2% at 0.5 mg/mL	Negative regulation of the expression of the genes VEGF, MMP-2, and MMP-9	[157]
Silver nanoparticles from ethanolic extract	A549	IC_50_ = 22 µg/mL at 48 h	ND	[158]
**Leek (*Allium ampeloprasum*)**				
Aqueous extract	MCF-7	Inhibition = 52.84% at 72 h at 50 μg/mL	ND	[159]
Methanolic extract		Inhibition = 40.86% at 72 h at 50 μg/mL		
Methanolic leaf extract	HCC (Liver cancer)	IC_50_ = 38.47 µg/mL at 72 h	Increased expression of the P53 gene	[160]
Methanolic leaf extract/metformin		IC_50_ = 1.36 µg/mL at 72 h	ND	
Silver nanoparticles from aqueous leaf extract	HT144 (Lung cancer)	IC_50_ = 125 µg/mL at 24 h	ND	[161]
	SKMEL2 (Lung cancer)	IC_50_ = 164 µg/mL at 24 h		
	WM266-4 (Lung cancer)	IC_50_ = 180 µg/mL at 24 h		
	IPC-298 (Lung cancer)	IC_50_ = 149 µg/mL at 24 h		
Gold nanoparticles from aqueous extract	MDA-MB-231	IC_50_ = 483.9 µg/mL	ND	[162]

ND: Not determined.

**Table 9:** In vitro anticancer activity of extracts and nanoparticles derived from Chenopodiaceae (beetroot, spinach, and chard).

Product Used	Cell Line	In Vitro Activity	Mechanism of Action	Reference
**Beet (*Beta vulgaris* L.)**				
Ethanolic extract of root	A549	Inhibition = 22% at 800 µg/mL	ND	[184]
Copper oxide nanoparticles from beetroot extract	A549	IC_50_ = 25 µg/mL	Apoptosis, cell cycle arrest in the G2/M phase	[185]
Aqueous extract of husk flour	MCF-7	LC_50_ = 16.6–22.6 mg/mL	ND	[186]
	MDA-MB-231	LC_50_ = 7.9–20.1 mg/mL		
Hydroalcoholic extract of roots	Caco-2	IC_50_ = 107.0 µg/mL at 48 h	Positive regulation of the expression of pro-apoptotic genes BAD, Fas-R, Caspase-3, Caspase-8, and Caspase-9. Decreased expression of Bcl-2	[187]
	HT-29	IC_50_ = 92.0 µg/mL at 48 h		
Beetroot fermented with water kefir	HepG2	Inhibition = 77.72% at 24 h	Apoptosis	[188]
**Spinach (*Spinacia oleracea* L.)**				
Hydroalcoholic extract	HT-29	Inhibition = ~80% a 500 µM at 48 h	Increased levels of intracellular endogenous reactive oxygen species when tested at higher doses	[189]
Normoxic leaf extract	HT29	Inhibition = 59.56% at 0.5 g eq. FW mL^−1^ at 12 h	ND	[190]
Hypoxic leaf extract		Inhibition = 92.3% a 0.5 g eq. FW mL^−1^ at 12 h		
75% ethanolic extract	K562	Inhibition = 88.9% at 10 mg/mL	ND	[191]
80% ethanolic extract of leaves	HeLa	IC_50_ = 13.80 µg/mL	Apoptosis	[192]
**Chard (*Beta vulgaris* L. var. *cicla* o *flavescens*)**				
Phenolic extract fraction of the leaves	MCF-7	IC_50_ = 9.1 µg/mL	Antimitotic activity inhibiting one step of the DNA synthesis pathway without involving multiple targets	[193]
Ethyl acetate extract from seeds	RKO (Poorly differentiated colon carcinoma)	IC_50_ = 32 µg/mL	Apoptosis, increased number of cells in the G1 phase and reduced number of cells in the S phase	[194]
Swiss chard extract after irrigation and fertilization	MCF-7	IC_50_ = 20.76 µg/mL at 48 h	ND	[195]
Untreated Swiss chard extract		IC_50_ = 23.33 µg/mL at 48 h		

ND: Not determined.

## Data Availability

No new data were created or analyzed in this study. Data sharing is not applicable to this article.

## Abbreviations

The following abbreviations are used in this manuscript:

IC_50_	Mean Maximum Inhibitory Concentration
MTT	3-(4,5-dimethylthiazol-2-yl)-2,5-diphenyltetrazolium bromide
LC_50_	Median lethal concentration
EC_50_	Average Effective Concentration
ND	Not determined
HPLC	High-performance liquid chromatography
